# A translational approach to ventilator associated pneumonia

**DOI:** 10.1186/2001-1326-3-26

**Published:** 2014-07-28

**Authors:** Ornella Piazza, Xiangdong Wang

**Affiliations:** 1 Università di Salerno, Baronissi (SA), Italy; 2 Fudan University, Shanghai, China; 3 Zhongshan Hospital, Fudan University, Shanghai, China

**Keywords:** VAP, Tracheobronchitis, Biomarkers, sTREM, Procalcitonin

## Abstract

The management of Ventilator Associated Pneumonia (VAP) presents many difficulties because of the heterogeneity of the disease; the way the immunocompromised host and the aggressive ICU environment interact is only partially discovered, the available biomarkers for diagnosis are not sufficient to ensure prompt differentiation between sick patients and patients at risk, the microbiological cultures require invasive techniques and time consuming methods. A translational medicine and bio-informatics approach can enable the identification of the main players of pathology, which may represent novel therapeutic targets or biomarker candidates. Analysis of proteome i.e. allows to individuate proteins that act as biomarkers, for patient-centered research strategies. Similarly, the genomic approach has proved useful to individuate those patients who are prone to develop VAP, and, in the future, we could be able to immunomodulate their responses to save them from nosocomial infections.

## Introduction

VAP (Ventilator Associated Pneumonia) is classically defined as a hospital-acquired pneumonia developing 48 hours or later after the beginning of mechanical ventilation.

Since VAP diagnosis founded on radiographic findings of pneumonia, which have intrinsic variability in technique, interpretation, and reporting, and on clinical signs and symptoms- that are subjective- in 2011 a Working Group of the CDC (Centers for Disease Control and Prevention) proposed a new approach to surveillance for Ventilator-Associated Events (VAE). According to the new CDC definition algorithm, VAP is an Infection-related Ventilator-Associated Complication (IVAC) occurring after 3 days of mechanical ventilation and 2 days before or after the onset of worsening oxygenation, if purulent respiratory secretions with positive cultures or objective signs of respiratory infection have been found [1]. The need for a new and efficacious operative definition is comprehensible, since VAP remains one of the commonest nosocomial infections in the intensive care unit (ICU), affecting 8% to 28% of patients receiving mechanical ventilation [2]. Patients who develop a VAP have significantly more ventilator days, hospital days, and antibiotic days and higher hospital mortality than patients who have not this condition. The hazard for mortality is calculated about 7.2 (95% CI 5.1 - 10.3) for VAP identified by prospective surveillance [3]. Thanks to the generic improvements in critical care [4], mortality following VAP decreased slightly over time. Even if the “ventilator bundle” (4 items to prevent respiratory complications: stress ulcer prophylaxis, deep venous thrombosis prophylaxis, head-of-bed elevation, daily sedation vacation with weaning assessment), introduced by the Institute for Health Care Improvement, has been partially accepted, there is little evidence of its efficacy up to now [5] and no specific intervention has been yet individuated to eradicate VAP. Over the past decade, the accuracy of initial antibiotic treatment has been manifest as one of the most consistent prognostic factor: early aggressive therapy with adequate broad-spectrum regimens against the likely pathogens is associated with lower mortality rates and shorter length of hospital stay (LOS). Patients receiving inadequate antibiotic therapy compared with patients receiving adequate antibiotic therapy reported a significantly longer hospital LOS: this was calculated as plus 16.4 days (p = 0.009) for VAP [6]. There is general agreement that the causative agent should be identified at the onset and intensely treated in order to achieve long-lasting remission but prompt diagnosis and individuation of pathogens are still difficult. Diagnostic methods with good sensitivity already exist for VAP: they are the simple clinical criteria, but a biomarker with high specificity it is needed to guide the clinician towards the sources of infection: increasing the specificity of any screening test, the fewer false positive would reduce the cost of antibiotics and invasive diagnostic strategies.

While its is clear the importance of a rapid causative diagnosis, it remains unknown why some intubated patients remain infection free while other develop VAP and other tracheobronchitis [7]. Ventilator-associated tracheobronchitis (VAT) is very common in intubated critically ill patients. This infection represents an intermediate process between colonization of lower respiratory tract and VAP. VAT is characterized by increased purulent sputum production and lower respiratory tract inflammation resulting in difficult weaning and prolonged duration of mechanical ventilation.

This area of controversy about the interaction between the patient and his/her intensive care environment is extremely relevant, since the rapid individuation of subject prone to infection should involve the aggressive treatment while patients who have mild and indolent disease need to be identified but not over treated. The role of the underlying disease should be considered as dominant and the primary endpoint in VAP management should ideally be prevention. Areas timely for developing research are strategy for VAP prevention, individuation of biomarkers for early diagnosis and subgroups identification, methods to develop and validate multi-drug resistant pathogens detection on bronchoalveolar lavage (BAL) or serum. We will expose the relevant result of recent clinical translational works that aim to identify novel pathways in VAP diagnosis and treatment.

### Biomarkers for VAP diagnosis

As already said, clinical criteria for VAP diagnosis present high sensibility but low specificity. Comorbidities and other concomitant conditions as leukocytosis may influence the evaluation of clinical data; fever in ICU patients is extremely common and radiologic criteria is often misleading. The Clinical Pulmonary Infection Score (CPIS) takes into account some easily available parameters: fever, leukocytosis, tracheal aspirates, oxygenation, radiographic infiltrates, and may increase the specificity of diagnosis when the sum of individual scores is higher than 6. The initial data, published by Plugin et al. [8] were not confirmed later in larger studies and, to increase the CPIS specificity, the semi-quantitative cultures of tracheal aspirates with Gram stain was introduced, still with limited efficacy [9]. The weak point of CPIS is probably the inter-individual variability, since a subjective evaluation is required when we are judging the quality of tracheal secretion (purulent/not purulent) and the presence of infiltrate at chest ray.

Since clinical criteria are not specific enough, the need for a simple clinical tool for the diagnosis of VAP is evident. A perfect biomarker (not expensive, not invasive, which helps to avoid excess antibiotic use and assist in the conduct of clinical research) is still lacking, but various biological elements have been proposed, among which Procalcitonin, Pentraxin3, and Soluble Triggering Expressed on Myeloid Cells Type 1 are the most cited in the literature.

Procalcitonin (PTC) is a pro-hormone, precursor of Calcitonin. PTC is synthesised in virtually all organs and in macrophages in response to pro-inflammatory stimuli, particularly stimuli of bacterial origin. In a metanalysis of 7 studies (373 patients, 434 episodes), Sotillo- Diaz et al. [10] found that high plasma PCT levels were associated to an increased risk of suffering ventilator-associated pneumonia (OR: 8.39; 95% CI: 5.4-12.6). Sensitivity and specificity were 76% (69–82) and 79% respectively. Even if these values are fairly acceptable, we should also consider that the CPIS score in selected population (i.e. brain injury) [11] scores even better: 97% sensitivity and 100% specificity and question the role of PCT as unique screening test for VAP early diagnosis [12].

The diagnosis of VAP is not the only field of application of PTC: it could be used for prognosis and to follow antibiotic therapy. Using the combination of PCT plus CPIS may be more powerful than PTC by itself: Su et al. [13] in 2012 demonstrated that procalcitonin levels plus the CPIS score was reliable for prognostic assessment (28-day survival), in sepsis patient with VAP.

Assuming the patients meet the clinical criteria for VAP, the PTC level could guide the duration of antibiotic therapy, allowing a safe individualised strategy for VAP. Pugh et al. [14] reviewed three RCTs which investigated strategies to reduce antibiotic exposure on the basis of procalcitonin: the experimental strategy advocated discontinuing antibiotic therapy when the serum PTC level was below a cut-off value (0,5 or 0.25 μg/L, depending on the investigators) or fell to less than 20% of the level on Day 0. Duration of antibiotic therapy was significantly shorter in patients treated according to PTC-guided strategy than for patients allocated to standard therapy (durations of therapy for the procalcitonin groups 9.2 days versus 12.1 days).

Taking the data all together, PTC looks useful but not unequivocal as a biomarker for VAP diagnosis. The explanation could be that PTC is a part of pro-inflammatory response of the immune system and it could be seen as an assistant to host, in recognition of invasion by bacteria, discriminating viral from bacterial infections. Nevertheless this hypothesis is just speculative and we need to understand how PTC really “works” during VAP.

Pentatraxin 3 (PTX3) is a protein targeted to inflammation and has been proposed as an alternative biomarker for VAP diagnosis. PTX3 is a member of the long pentraxin subfamily while the C-reactive protein (CRP) is the prototype of short pentraxin and has been widely used, with limited diagnostic and prognostic specificity in infectious diseases.

PTX3 is released by mononuclear phagocytes, neutrophils, epithelial and endothelial cells in response to inflammatory signals; PTX3 recognizes microbial molecules, activates the classical pathway of complement and facilitates recognition by macrophages and dendritic cells. Lin et al. [15] performed the measurement of PTX3 and CRP levels in plasma from 136 consecutive patients receiving mechanical ventilation > 48 hours in a prospective single centre study. PTX3 > 16.43 ng/ml showed specificity of 74.0% and sensitivity of 68.6%, not superior to CRP as a biomarker to diagnose VAP. The authors admit some limitations of this single centre study and propose a larger trial, which at the moment is lacking. Nevertheless, PTX3 is not a specific marker for bacterial infection. Elevated plasma PTX3 concentrations are seen in various inflammatory conditions, which can be concomitant to VAP diagnosis, making PTX3 a marker not enough specific for this complication.

Triggering receptor expressed on myeloid cells-1 (TREM) is a glycoprotein member of the immunoglobulin superfamily. TREM expression is up-regulated in the presence of extracellular bacteria and fungi and some inflammatory conditions. In response to infection, soluble TREM-1 (sTREM-1) can then be measured in body fluids, while sTREM-1 levels are not detectable at baseline in normal individuals.

In Palazzo et al. paper, sTREM-1 levels did not discriminate VAP when compared to quantitative cultures of bronchoalveolar lavage (commonly used as the gold standard) [16]. Using a cutoff value of 204 pg/mL for BALF sTREM-1 levels resulted in sensitivity of 79% and specificity of 23%.

In conclusion, up to now no biomarker has yet improved our clinical capacity to diagnose VAP: biologic markers have been suggested to improve recognition of patients with true infection and facilitate decisions of whether or not to treat. Unfortunately, neither PCT, PTX3 or sTREM-1 fulfill all expectations.

In the recent years, it is been recognized that the multivariate model is more promising than a single biomarker for risk assessment of disease. This multi-biomarker approach has progressed by recent advances in clinical proteomics. Proteomics is a branch of biotechnology concerned with applying the techniques of molecular biology, biochemistry, and genetics to analyzing the structure, function, and interactions of the proteins produced by the genes of a particular cell, tissue, or organism, with organizing the information in databases, and with applications of the data (as in medicine or biology) [17]. In 2008 Lu et al. published the first global description of the BAL proteome from patients with VAP: they identified 206 proteins, defining the global proteome map of BAL. Four selected proteins (gelsolin, serum amyloid P-component, vitamin D-binding protein and pyruvate kinase) [18] were significantly higher in BAL from patients with VAP (p < 0.05). Applying a modern proteomic approach Nguyen et al. [19] identified a proteomic “signature” that discriminated VAP(+) from VAP(−) patients in Acute Lung Injury patients: it is constituted by three BAL proteins: S100A8, lactotransferrin (LTF), and actinin 1 (ACTN1).

The proteomic approach needs computational analyses to became really powerful and study the proteome during VAP. This integrative methodology is a strategy to differentiate clinically relevant subsets of patients.

Metabolomics is the quantitative analysis of a large number of low molecular mass metabolite, substrates or products in metabolic pathways. It’s important to note that Rogers et al. recently published a metabolomic study in critically ill patients [20]: they formed a metabolomic network of 7 metabolites associated with death (gamma-glutamylphenylalanine, gamma-glutamyltyrosine, 1-arachidonoylGPC(20:4), taurochenodeoxycholate, 3-(4-hydroxyphenyl) lactate, sucrose, kynurenine). This network achieved a 91% AUC predicting 28-day mortality. Similar studies are still lacking in the field of VAP but would be a very practical and interesting translational approach application.

### Prompt identification of VAP caused by MDR bacteria

Once the clinical suspicion of VAP is consolidated, selection of the appropriate antimicrobial therapy is the most urgent and following step. Quantitative culture of bronchoalveolar lavage (BAL) has the highest sensitivity and specificity to diagnose VAP and differentiate true infection from colonisation or inflammation. The CDC recommends that quantitative cultures be performed on these specimens, using ≥ 10^4^ CFU/ml to designate a positive culture (Table 1). Bronchoscopy - necessary to perform BAL- is invasive and requires specialised skills. All culture-based methods may require up to 48 hours for microbial identification: while waiting for microbiology results a broad spectrum antibiotic therapy has to be started. Causative agent may be the most various: Staphylococcus aureus, Pseudomonas aeruginosa and Enterobacteriaceae represent the most frequent pathogens in VAP [21,22]. As a result of the overuse of broad-spectrum antimicrobials such as the carbapenems, strains of Acinetobacter, Enterobacteriaceae, and Pseudomonas aeruginosa susceptible only to polymyxins and tigecycline have emerged as important causes of VAP [23]. Definition of VAP implies that the patient has been intubated (or has a tracheostomy) for more than 48 hours. A further differentiation can be made, considering the time of onset: within 96 hours from intubation (early onset VAP) or after 96 hours of intubation (late onset VAP). This differentiation is important to discriminate the potential causative pathogens, since the late onset VAP is very frequently due to multi-drug resistant (MDR) agents. The clinician will start therapy using broad-spectrum antibiotics and stop it if the diagnosis of infection becomes unlikely. The need to accurately diagnose VAP so that appropriate discontinuation or de-escalation of antimicrobial therapy can be initiated (to reduce the antimicrobial pressure) is evidently essential. The second step is then using narrower spectrum antibiotics, once the etiologic agent is identified, taking into consideration the pharmacokinetic and pharmacodynamic properties of the agent selected. Provided that initial therapy was appropriate, the clinical course appears favourable, and microbiologic data do not point to a very difficult-to-treat microorganism, the physician could switch to mono-therapy on days 3–5. But, what if the initial therapy is not appropriate or if a therapy is started but is inefficacious against drug resistant bacteria?

**Table 1:** **CDC Algorithm for VAP diagnosis [**[1]**]**

**1= Purulent respiratory secretions AND one of the following:**	**2= One of the following (without requirement for purulent respiratory secretions):**
• Positive culture of endotracheal aspirate, ≥ 10^5^ CFU/ml *	• Positive pleural fluid culture
• Positive culture of bronchoalveolar lavage, ≥ 10^4^ CFU/ml*	• Positive lung histopathology
• Positive culture of lung tissue, ≥ 10^4^ CFU/ml*	• Positive diagnostic test for Legionella spp.
• Positive culture of protected specimen brush, ≥ 10^3^ CFU/ml*	• Positive diagnostic test on respiratory secretions for influenza virus, respiratory syncytial virus, adenovirus, parainfluenza virus

On or after calendar day 3 of mechanical ventilation and within 2 calendar days before or after the onset of worsening oxygenation, criteria 1 or 2 is met (*or equivalent semi-quantitative result).

A clear example comes from the difficult management of VAP caused by Acinetobacter baumannii, a resilient Gram-negative cocco-bacillus, responsible of serious nosocomial infection, among which is included VAP [24]. Transmission of A. baumannii in association with contamination of hospital equipment or cross-transmission among patients is easy and common. This spread is linked to the capacity of bacteria to survive in dry environments and their need for simple growth conditions and to withstand disinfection and to persist in the hospital environment. A. baumannii shows a broad spectrum of resistance to antimicrobial agents: carbapenems have been reported as the most appropriate choice for the treatment, but in A. baumannii resistance to carbapenems may be conferred by some carbapenemases (metalloenzymes) that hydrolyze imipenem and meropenem. In case of CRAB (carbapenem-resistant A. baumannii), colistin is the drug of choice to cure the infection even if it has severe collateral effects and a large rate of failures [25]. Then, A. Baumanni infections need to be rapidly identified, in order to treat the pathogens and cure the patients but also limit the spread, which can cause a micro-epidemic within the ICU. Nevertheless, resistant A. baumannii needs to be promptly isolated and the resistant strains have to be identified to allow the use of colistin or combination of drugs since the first day of VAP diagnosis, not waiting for the time (3–4 days, depending on the institution) necessary for obtaining the results of culture and sensitivity. The importance to start the correct regimen immediately is evident in a serious and potentially epidemic disease as VAP caused by MDR organism but the clinical routine has not yet the tools to get simplification of the causative diagnosis. Promising new techniques and the application of translational methods to daily practice may overcome some difficulties. Real-time PCR, hybridization and mass spectrometry-based platforms are methods providing quantitative results, which can support organism identification and detect bacterial resistance directly from clinical specimens [26].

Quantitative polymerase chain reaction (qPCR) i.e. may reduce the time for microbiological diagnosis, allowing to start immediately the right antibiotics and avoiding unnecessary overtreatment. Kwon et al. tried qPCR for etiologic diagnosis of Methicillin-Resistant Staphylococcus aureus (MRSA) pneumonia on bronchoalveolar lavage or bronchial washing samples [27]. Molecular identification of MRSA was based on the presence of the mecA and femA-SA gene, with the absence of the femA-SE gene. The sensitivity and specificity of this assay were 88.9% and 88.9% respectively (compared with quantitative cultures). Applying this assay to BAL analysis certainly improves the diagnosis but it does not avoid the use of invasive technique as bronchoscopy. To identify pathogen-derived volatile biomarkers in breath would be a non- invasive method to diagnose VAP. Filipiak et al. made in vitro experiments with Staphylococcus aureus and Pseudomonas aeruginosa (bacteria most frequently found in VAP patients) to investigate the release or consumption of volatile organic compounds by gas chromatography mass spectrometry (GC-MS). The Authors concluded that: “the detection and perhaps even identification of bacteria could be achieved by determination of characteristic volatile metabolites, supporting the clinical use of breath-gas analysis as a non-invasive method for early detection of bacterial lung infections” [28]. Chromatography for microbiological definition appears as a very interesting application of a translational technique to VAP microbiological diagnosis.

### Pathogenesis of VAP and its prevention in a genomic perspective

The relevance of VAP pushed the development of a national programme in the USA to reduce its incidence [5]. Many efforts have been proved inefficacious or controversial (e.g. selective digestive decontamination) till now. Nevertheless, counteracting the pathogenetic pathways and limiting the risk factors sounds a reasonable option. The first measure to reduce VAP is to limit exposure to mechanical ventilation by preferring non-mechanical ventilation when possible and limiting its duration. Other prevention practices aim at reducing airways colonisation (such as hygiene before manipulating airways and oral care decontamination using chlorhexidine), or preventing aspiration (e.g. by nursing in the semirecumbent position, or maintaining a sufficient cuff pressure).

**Figure 1:**
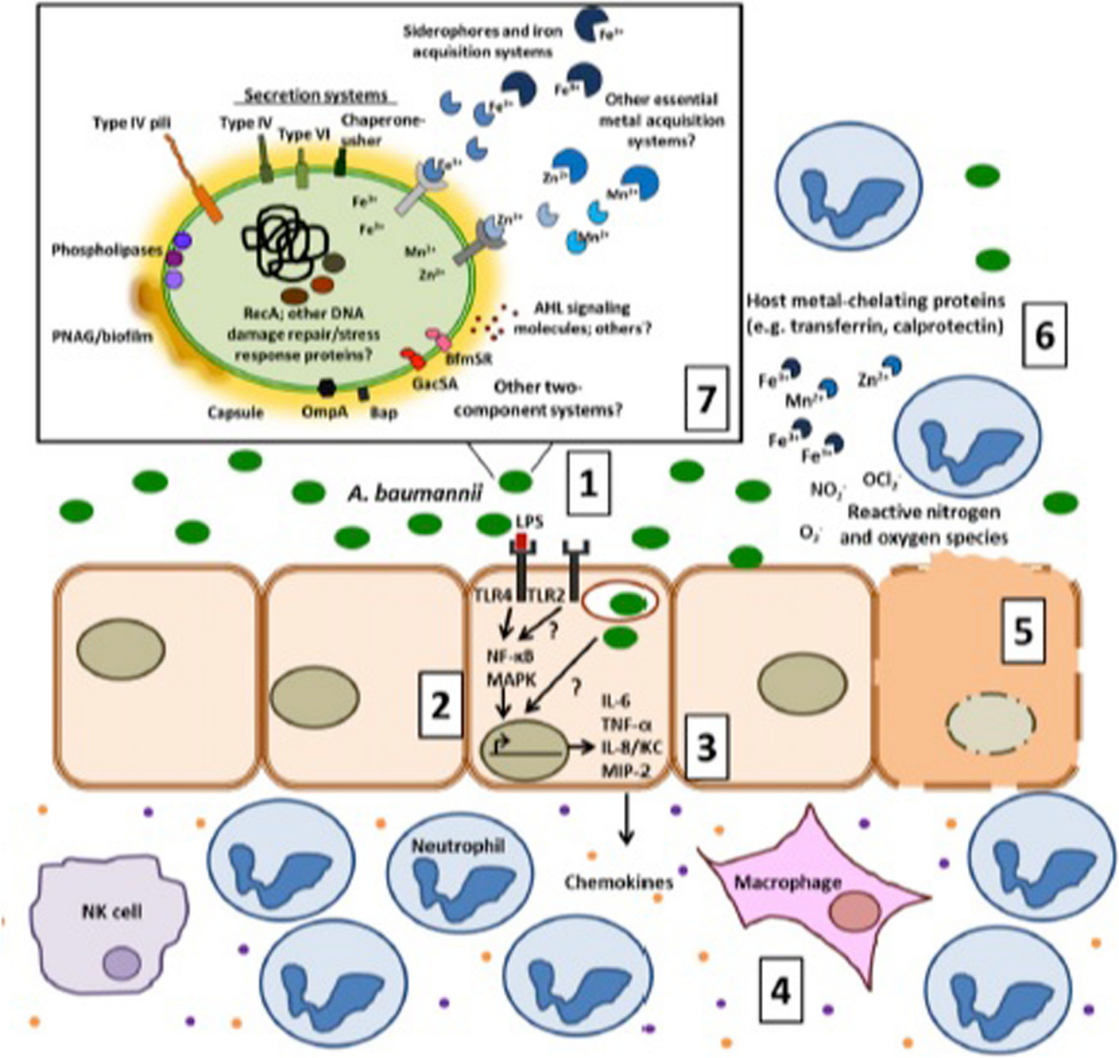
**From Mortensen**[29]**with permission: summary of the dynamic interplay between A. baumannii and the host.** (1) A. baumannii can adhere and invade host cells, leading to stimulation of the pro-inflammatory immune response. (2) The inflammatory response is initiated by TLR4 recognition of LPS which then activates MAPK and NF-κB pathways. TLR2 is also reported to detect A. baumannii. (3) Activation of these receptor proteins leads to subsequent transcription and secretion of pro-inflammatory mediators such as cytokines IL-6 and TNF-α and chemokines KC/IL-8 and MIP-2. (4) These chemokines recruit granulocytes and lymphocytes that are required for controlling infection. (5) Following A. baumannii infection host cells also undergo apoptosis. (6) Other host defences include nutritional immunity, ROS/RNS production, and antimicrobial peptides. (7) In response to the host environment, A. baumannii expresses several virulence factors implicated in pathogenesis, which are displayed in the inset of the figure. The illustration depicts those proteins and molecules that are functionally characterized and those that are predicted to be expressed. The question marks designate areas in which there are significant gaps in our knowledge.

Colonisation of the upper airways is probably the main risk factor for VAP: it is extremely frequent and rapidly acquired in ICU but not all the colonised patients develop any clinical significant infection, neither all the tracheobronchitis (VAT) become VAP. Normally, bacterial pathogen-associated molecular patterns (PAMPs) are detected by innate immune receptors on host cells, whose intracellular signalling pathways promote transcription, processing and secretion of inflammatory mediators [29]. The immune response is principally dependent on how the host interacts with a given pathogen (Figure 1).

Martin Loaches et al. compared VAP/VAT gene expression using genome-wide oligonucleotide microarrays [7]. Comparison of gene expression profiles in the pre-infection period revealed 5595 genes expressed differentially between VAP and VAT. The Authors reported that patients who developed VAP had immune impairment in the pre-infection period, with a lower expression of genes involved in the complement system, compared to patients developing simple tracheobronchitis.

**Figure 2:**
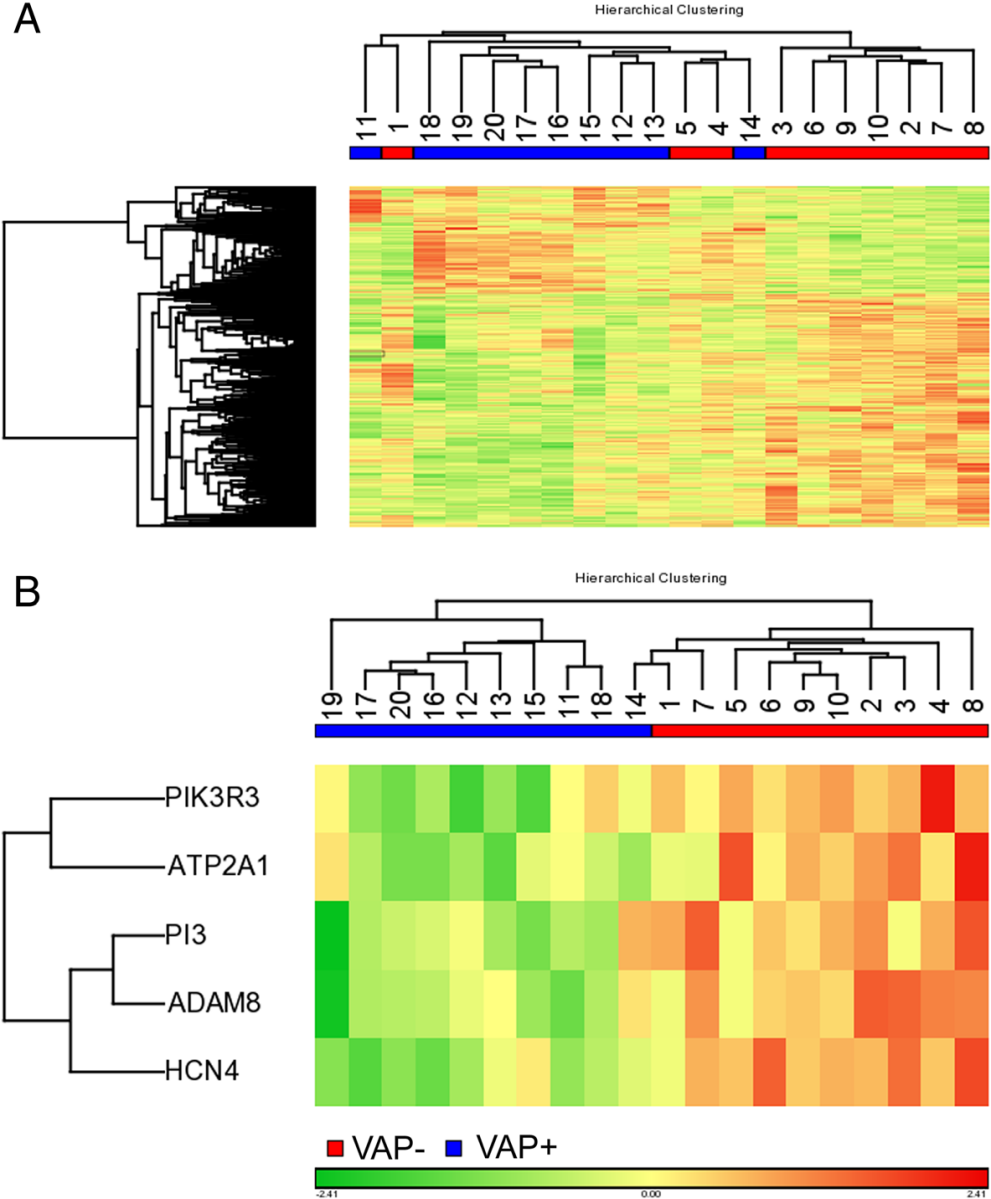
**From Swanson with permission: hierarchical clustering of VAP − and VAP + patients. (A)** Hierarchical clustering of 810 differentially expressed genes in patients that went on to develop ventilator-associated pneumonia (blue) and those that did not (red). **(B)** Hierarchical clustering with the five genes that were common to all sets used in the cross validation tests.

Swanson et al. in 10 trauma patients that later developed VAP, found that gene expression profiles of lipopolysaccharide stimulated blood cells were different from those expressed in controls who did not develop VAP and showed 5 genes (PIK3R3, ATP2A1, PI3, ADAM8, and HCN4) common to all significant gene sets used in the cross validation tests [30]. After LPS stimulation of whole blood, the expression of these five genes was down regulated in patients who later suffered from VAP. Hierarchical clustering using these five genes accurately categorized 95% of patients and PCA visualization demonstrated two discernable groups, VAP + and VAP − (Figure 2). Of the five genes identified, three appear to have a role in host response to infection while HCN4 and ATP2A1 are not directly linked with infection. The environment of the immune-compromised host and the contribution of the anti-inflammatory response to controlling damaging inflammation have important implications to the pathogenesis that we are beginning to appreciate. Immunomodulatory therapeutics in these patients with compromised immune status may be a beneficial strategy for treatment of nosocomial infections.

## Review and conclusion

**Figure 3:**
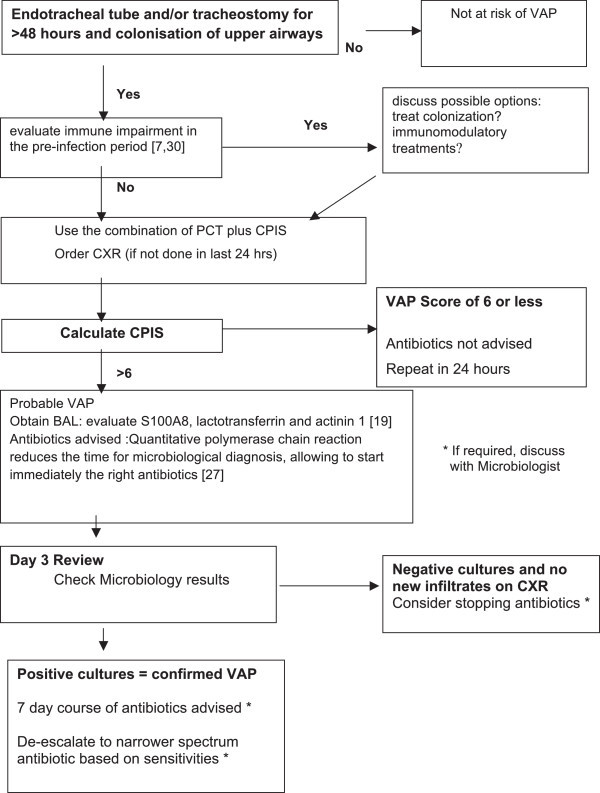
A tentative flow chart to introduce step by step the alternatives from translational medicine step by step.

Ventilator Associated Pneumonia (VAP) is one of the most common nosocomial infections in ICU presenting with not specific symptoms and clinical signs. Too many VAP patients still receive inadequate initial antibiotics even if it is well known that the incidence of MDR pathogen infections is on the rise in ICU. VAP is one of the major sites for emergence of MDR pathogens because subtherapeutic antibiotic concentrations in the lung necessitate longer duration of therapy, thereby favoring selection of resistant bacteria. The choice of the initial empiric antibiotic is challenging; the administration of “broad spectrum” antibiotics may be the safest decision but is associated to increased resistance rate and failure of therapy. Combined antibiotics regimens or more risky antibiotics as colistin may be the only available choice i.e. when MDR *Acinetobacter* spp. is suspected. The most practical solution, at the moment, seems to reduce the time of empiric therapy at its minimum, starting as soon as possible a short course of focused antibiotics. A short course of antibiotic treatment (7–8 days) is effective at successfully clearing the infection, reducing the duration of antibiotic administration, and decreasing antibiotic resistance without affecting in-hospital mortality, ventilator days, or hospital length of stay when compared with a 10-day to 15-day course but currently, there is not enough data to support further shortening the duration of antibiotic treatment in patients with culture proven VAP.Although our insight has significantly increased over the past years, a translational approach, with application of genomics, proteomics and metabolomics methodologies to this severe clinical problem is required to better understand the disease and hypothesize new therapeutic strategies. Translational approach seems to be able to boost the necessary knowledge of VAP in several ways (Figure 3). The vast number of measurements that could becomes available for VAP will require powerful informatics methods for organizing the data. We should look for deeper interactions among basic science researchers and clinicians to push on translational approach even in the newest fields as nutribolomics, which could provide new insight and possibility for the cure of VAP.

## Competing interest

The authors declare that they have no competing interests.

## Authors’ contributions

OP conceived of the study, and drafted the manuscript. XW conceived of the study and helped to draft the manuscript. Both authors read and approved the final manuscript.

## References

[B1] Centers for Disease Control and Prevention **Ventilator-Associated Event (VAE)** 2014 3 1 46 http://www.cdc.gov/nhsn/pdfs/pscManual/10-VAE_FINAL.pdf

[B2] Di Bonito M Caiazzo S Iannazzone M Miccichè V De Marco G De Robertis E Tufano R Piazza O Prognostic differences between VAP from acinetobacter baumanii and VAP from other microorganisms Transl Med UniSa 2012 3 15 21 23905048 PMC3728786

[B3] Klein Klouwenberg PM van Mourik MS Ong DS Horn J Schultz MJ Cremer OL Bonten MJ on behalf of the MARS consortium Electronic implementation of a novel surveillance paradigm for ventilator-associated events: feasibility and validation Am J Respir Crit Care Med 2014 3 8 947 955 24498886 10.1164/rccm.201307-1376OC

[B4] Rosenberger LH Hranjec T McLeod MD Politano AD Guidry CA Davies S Sawyer RG Improvements in pulmonary and general critical care reduces mortality following ventilator-associated pneumonia J Trauma Acute Care Surg 2013 3 2 568 574 23354252 10.1097/TA.0b013e3182789312 PMC3558924

[B5] Croce MA Brasel KJ Coimbra R Adams CA Jr Miller PR Pasquale MD McDonald CS Vuthipadadon S Fabian TC Tolley EA National trauma institute prospective evaluation of the ventilator bundle in trauma patients: does it really work? J Trauma Acute Care Surg 2013 3 2 354 360 23354225 10.1097/TA.0b013e31827a0c65

[B6] Piskin N Aydemir H Oztoprak N Akduman D Comert F Kokturk F Celebi G Inadequate treatment of ventilator-associated and hospital-acquired pneumonia: risk factors and impact on outcomes BMC Infect Dis 2012 3 268 23095664 10.1186/1471-2334-12-268 PMC3511218

[B7] Martin-Loeches I Papiol E Almansa R López-Campos G Bermejo-Martin JF Rello J Intubated patients developing tracheobronchitis or pneumonia have distinctive complement system gene expression signatures in the pre-infection period: a pilot study Med Intensiva 2012 3 4 257 263 22301068 10.1016/j.medin.2011.10.009

[B8] Pugin J Auckenthaler R Mili N Janssens JP Lew PD Suter PM Diagnosis of ventilator-associated pneumonia by bacteriologic analysis of bronchoscopic and nonbronchoscopic “blind” bronchoalveolar lavage fluid Am Rev Respir Dis 1991 3 5 Pt 1 1121 1129 2024824 10.1164/ajrccm/143.5_Pt_1.1121

[B9] Zilberberg MD Shorr AF Ventilator-associated pneumonia: the clinical pulmonary infection score as a surrogate for diagnostics and outcome Clin Infect Dis 2010 3 Suppl 1 S131 S135 20597663 10.1086/653062

[B10] Sotillo-Díaz JC Bermejo-López E García-Olivares P Peral-Gutiérrez JA Sancho-González M Guerrero-Sanz JE Role of plasma procalcitonin in the diagnosis of ventilator-associated pneumonia: Systematic review and metaanalysis Med Intensiva 2014 3 6 337 346 24035696 10.1016/j.medin.2013.07.001

[B11] Pelosi P Barassi A Severgnini P Gomiero B Finazzi S Merlini G d’Eril GM Chiaranda M Niederman MS Prognostic role of clinical and laboratory criteria to identify early ventilator-associated pneumonia in brain injury Chest 2008 3 1 101 108 18403669 10.1378/chest.07-2546

[B12] Zielińska-Borkowska U Skirecki T Złotorowicz M Czarnocka B Procalcitonin in early onset ventilator-associated pneumonia J Hosp Infect 2012 3 2 92 97 22552164 10.1016/j.jhin.2012.02.011

[B13] Su LX Meng K Zhang X Wang HJ Yan P Jia YH Feng D Xie LX Diagnosing ventilator-associated pneumonia in critically ill patients with sepsis Am J Crit Care 2012 3 6 e110 e119 23117911 10.4037/ajcc2012732

[B14] Pugh R Grant C Cooke RP Dempsey G Short-course versus prolonged-course antibiotic therapy for hospital-acquired pneumonia in critically ill adults Cochrane Database Syst Rev 2011 3 CD007577 10.1002/14651858.CD007577.pub2 21975771

[B15] Lin Q Fu F Shen L Zhu B Pentraxin 3 in the assessment of ventilator-associated pneumonia: an early marker of severity Heart Lung 2013 3 2 139 145 23273657 10.1016/j.hrtlng.2012.11.005

[B16] Palazzo SJ Simpson TA Simmons JM Schnapp LM Soluble triggering receptor expressed on myeloid cells-1 (sTREM-1) as a diagnostic marker of ventilator-associated pneumonia Respir Care 2012 3 12 2052 2058 22613763 10.4187/respcare.01703 PMC4432465

[B17] Anderson NL Anderson NG Proteome and proteomics: new technologies, new concepts, and new words” Electrophoresis 1998 3 11 1853 1861 9740045 10.1002/elps.1150191103

[B18] Lu YT Han CL Wu CL Yu TM Chien CW Liu CL Chen YJ Proteomic profiles of bronchoalveolar lavage fluid from patients with ventilator-associated pneumonia by gel-assisted digestion and 2-D-LC/MS/MS Proteomics Clin Appl 2008 3 9 1208 1222 21136918 10.1002/prca.200800069

[B19] Nguyen EV Gharib SA Palazzo SJ Chow YH Goodlett DR Schnapp LMProteomic profiling of bronchoalveolar lavage fluid in critically ill patients with ventilator-associated pneumonia PLoS One 2013 3 3 e58782 doi:10.1371/journal.pone.0058782 23505561 10.1371/journal.pone.0058782 PMC3591362

[B20] Rogers AJ McGeachie M Baron RM Gazourian L Haspel JA Nakahira K Fredenburgh LE Hunninghake GM Raby BA Matthay MA Otero RM Fowler VG Rivers EP Woods CW Kingsmore S Langley RJ Choi AM Metabolomic derangements are associated with mortality in critically ill adult patients PLoS One 2014 3 1 e87538 24498130 10.1371/journal.pone.0087538 PMC3907548

[B21] Barbier F Andremont A Wolff M Bouadma L Hospital-acquired pneumonia and ventilator-associated pneumonia: recent advances in epidemiology and management Curr Opin Pulm Med 2013 3 3 216 228 23524477 10.1097/MCP.0b013e32835f27be

[B22] Cataldi M Sblendorio V Leo A Piazza O Biofilm-dependent airway infections: a role for ambroxol? Pulm Pharmacol Ther 2014 3 2 98 108 24252805 10.1016/j.pupt.2013.11.002

[B23] Lambiase A Rossano F Piazza O Del Pezzo M Catania MR Tufano R Typing of pseudomonas aeruginosa isolated from patients with VAP in an intensive care unit New Microbiol 2009 3 3 277 283 19845110

[B24] Lambiase A Piazza O Rossano F Del Pezzo M Tufano R Catania MR Persistence of carbapenem-resistant acinetobacter baumannii strains in an Italian intensive care unit during a forty-six month study period New Microbiol 2012 3 2 199 206 22707133

[B25] Aydemir H Akduman D Piskin N Comert F Horuz E Terzi A Kokturk F Ornek T Celebi G Colistin vs. the combination of colistin and rifampicin for the treatment of carbapenem-resistant acinetobacter baumannii ventilator-associated pneumonia Epidemiol Infect 2013 3 6 1214 1222 22954403 10.1017/S095026881200194X PMC9151808

[B26] Lung M Codina G Molecular diagnosis in HAP/VAP Curr Opin Crit Care 2012 3 5 487 494 22889869 10.1097/MCC.0b013e3283577d37

[B27] Kwon SJ Jeon T Seo D Na M Choi EG Son JW Yoo EH Park CG Lee HY Kim JO Kim SY Kang J Quantitative PCR for etiologic diagnosis of methicillin-resistant staphylococcus aureus pneumonia in intensive care unit Tuberc Respir Dis (Seoul) 2012 3 3 293 301 23227069 10.4046/trd.2012.72.3.293 PMC3510279

[B28] Filipiak W Sponring A Baur MM Filipiak A Ager C Wiesenhofer H Nagl M Troppmair J Amann A Molecular analysis of volatile metabolites released specifically by staphylococcus aureus and pseudomonas aeruginosa BMC Microbiol 2012 3 113 22716902 10.1186/1471-2180-12-113 PMC3444334

[B29] Mortensen BL Skaar EP Host-microbe interactions that shape the pathogenesis of acinetobacter baumannii infection Cell Microbiol 2012 3 9 1336 1344 22640368 10.1111/j.1462-5822.2012.01817.x PMC3781221

[B30] Swanson JM Wood GC Xu L Tang LE Meibohm B Homayouni R Croce MA Fabian TC Developing a gene expression model for predicting ventilator-associated pneumonia in trauma patients: a pilot study PLoS One 2012 3 8 e42065 22916119 10.1371/journal.pone.0042065 PMC3419717

